# Quality of Life Among Residents of General Surgery Residency Training Program in Saudi Arabia: A Nationwide Study

**DOI:** 10.7759/cureus.42050

**Published:** 2023-07-17

**Authors:** Jubran J Al-Faifi, Rashad Nassar, Rayan Alharbi, Abdulaziz M Junid, Abdulmajeed Alarfaj

**Affiliations:** 1 College of Medicine, Imam Mohammad Ibn Saud Islamic University, Riyadh, SAU

**Keywords:** medical education, general surgery residents, kingdom of saudi arabia (ksa), general surgery quality of life, work-related quality of life (wrqol) scale, resident surgeon, training program, general surgery

## Abstract

Introduction

The General Surgery Residency Training Program is known to be one of the most challenging programs, which greatly impacts the resident’s quality of life (QoL) during their training years. Undertraining residents are usually the first providers of patients’ healthcare in medical facilities. They often get exposed to continuous pressure and stress, especially during long working hours.

Aim

This study aims to evaluate the quality of life (QoL) of general surgery residents in Saudi Arabia and investigate the personal and workplace determinants associated with the level of quality of life.

Subjects and methods

This cross-sectional study was conducted among general surgery residents in Saudi Arabia. A self-administered online questionnaire was distributed among the target residents. The questionnaire includes sociodemographic characteristics (e.g., gender, region of the training center, and residency level) and Work-Related Quality of Life (WRQoL) scale to measure the residents’ quality of life at work.

Results

Of the 239 residents, 64.9% were males, and 27.2% were resident level 1. Among WRQoL components, only home-work interface (HWI) (mean score: 9.87 out of 15 points) and general well-being (GWB) (mean score: 20.6 out of 30 points) had average ratings, while control at work (CAW), job and career satisfaction (JCS), stress at work (SAW), and working conditions (WCS) were classified as good. The overall WRQoL was deemed good (mean score: 81.3 out of 115 points). Being a female and practicing residency inside central region were the factors associated with better WRQoL. No significant differences were observed between WRQoL in terms of residency level, marital status, and previous visitation to a psychiatrist or psychologist (p>0.05).

Conclusion

Nearly one-third of the general surgery residents perceived their WRQoL as good. Female residents practicing in the central region demonstrated better quality of life as compared to the rest of the residents. Further research is needed to establish the level of WRQoL and its effect on general surgery residents during residency training.

## Introduction

The General Surgery Residency Training Program is known to be one of the most challenging programs, which has a huge impact on residents’ quality of life (QoL) during their training years [1]. In 2012, the World Health Organization defined quality of life (QoL) as the individual’s perception of their position in life in the context of culture, values, personal goals, expectations, standards, and concerns [2]. Many studies have demonstrated that undertraining residents are usually the first providers of patients’ healthcare in medical facilities, and they often get exposed to continuous pressure and stress, especially during long working hours.

In Japan, for example, a study evaluated the working environment and the amount of stress, and the relationship between long working hours and depression among first-year residents, which showed that most of the residents have depressive symptoms because of the long working hours. Factors such as sleep deprivation, peer pressure, and poor working environment may predispose to diminished or suboptimal quality of life among residents [3-6].

Until now, there is only one locally based study that has evaluated the quality of life among Saudi surgical field residents in 2019 in King Abdulaziz Medical City (Jeddah, Saudi Arabia), but there is no single study done specifically for Saudi general surgery residents in Saudi Arabia as a nationwide study. The aim of this study was to evaluate the quality of life among general surgery residents in Saudi Arabia and address the factors that are negatively affecting their quality of life [1].

## Materials and methods

Study design and setting

This is a cross-sectional study that depends on a self-administered questionnaire that was adopted from the Work-Related Quality of Life (WRQoL) scale. All registered general surgery residents in all five training regions in Saudi Arabia during the period from December 1, 2022, to May 30, 2023, have been invited electronically through emails and WhatsApp messages to voluntarily and anonymously participate in this study that assesses the quality of life among the Saudi general surgery training program trainees.

In the present study, we aimed to identify the necessary sample size for a population of 500 residents. To achieve a confidence level of 95% and a margin of error of 5%, we used the following formula: n = (Z^2 * p * (1-p)) / E^2. Based on this calculation, we determined that a sample size of 235 residents is required for our study.

We distributed the self-administered questionnaire to 299 residents, and 239 (80%) residents completed the questionnaire and were included in the data analysis.

Consent was obtained from all participants in this study. Professor Abdulaziz Al-Akaabba issued approval 454/2023, and this study was approved by the Institutional Review Board of the College of Medicine, Imam Mohammad Ibn Saud Islamic University (IMSIU-545/2023).

Study subjects

The study targeted all registered general surgery residents in Saudi Arabia during the period of six months (December 2022 to May 2023). The study included all general surgery residents, all levels of training, and any nationality who are training in Saudi Arabia in the General Surgery Residency Training Program and officially registered in the Saudi Commission for Health Specialties (SCFHS). The study has excluded non-general surgery residents and those who are not officially registered in the General Surgery Residency Training Program in SCFHS.

Research tools and procedures

The study depended on a self-reported questionnaire in which the questions were gathered specifically from the WRQoL scale. The WRQoL scale is a 23-item psychometric scale used to gauge the perceived quality of life of employees as measured through six psychosocial sub-factor. The WRQoL scale was used by individuals, organizations, and consultants as well as researchers as an aid to assessing and understanding the quality of working life of working people [7]. To collect data, the self-administered questionnaire was sent through emails and social media applications (Twitter, WhatsApp, and Telegram). All components of the questionnaire were clarified through hyperlinks, and if any question was not clear enough to the participant, the email addresses of the study representatives were included to facilitate communication and provide further information.

Questionnaire criteria

Residents’ quality of life has been assessed using the Work-Related Quality of Life (WRQoL) scale developed by Easton and Van Laar (2013) [7]. This is a 23-item scale questionnaire with 5-point Likert scale categories ranging from “strongly disagree” to “strongly agree” used to gauge the perceived quality of life of employees, which was composed of six components: control at work (CAW), home-work interface (HWI), general well-being (GWB), working conditions (WCS), job and career satisfaction (JCS), and stress at work (SAW). The level of WRQoL has been categorized using cutoff points (33% and 67%) taken from the study published by Almhizai et al. (2022) [6,7].

Data analysis

Frequency and percentage were used for the description of categorical variables including age and gender, while mean and standard deviation (SD) were used for continuous categories as the questionnaire outcomes. In the analysis of the WRQoL tool, all answers were coded as strongly disagree, disagree, neutral, agree, and strongly agree. For negative states, a reverse count was conducted. Then, questions were divided into six categories as provided by the questionnaire design, and for each category, the sum of scores was calculated. The results of each category and the total score were categorized as low, average, and good for scores of 33%, 67%, and up to 100%, respectively. T-test and Chi-test test were used for assessing the relation between demographic factors and quality of life where all statements with a p-value equal to or lower than 0.05 would be considered significant.

Statistical analysis

Statistical data were analyzed using the Statistical Package for the Social Sciences (SPSS) version 26 (IBM SPSS Statistics, Armonk, NY, USA). For categorical variables, frequency and proportion (%) were used. For continuous variables, mean ± standard deviation was utilized. The association between the overall WRQoL and the sociodemographic characteristics of the residents has been assessed using the Mann-Whitney Z-test. Statistical collinearity has been measured using the Shapiro-Wilk test and Kolmogorov-Smirnov test. The overall WRQoL follows the non-normal distribution. Therefore, the non-parametric test was applied. A p-value of 0.05 was taken as a cutoff point to determine statistical significance.

## Results

The sample consisted of 239 residents (64.9% male versus 35.1% female), and 45.2% were practicing at the central region training center. Of the participants, 27.2% were resident level 1, and 57.3% were unmarried. The prevalence of residents who had previous visits to a psychiatrist or a psychologist was 23.8%. Of them, 66.7% had follow-up visits (Table 1).

**Table 1:** Residents’ sociodemographic characteristics (n=239) SCFHS: Saudi Commission for Health Specialties

Study variables	Number (%)
Gender	
Male	155 (64.9%)
Female	84 (35.1%)
Region of training center SCFHS branch	
Central region	108 (45.2%)
Eastern region	36 (15.1%)
Northern region	24 (10%)
Southern region	49 (20.5%)
Western region	22 (9.2%)
Residency level	
R1	65 (27.2%)
R2	64 (26.8%)
R3	53 (22.2%)
R4	33 (13.8%)
R5	24 (10%)
Marital status	
Single	137 (57.3%)
Married	80 (33.5%)
Divorced	22 (9.2%)
Have you ever been to a psychiatrist or a psychologist before?	
Yes	57 (23.8%)
No	182 (76.2%)
If “yes,” do you have follow-up visits? (n=57)	
Yes	38 (66.7%)
No	19 (33.3%)

**Figure 1:**
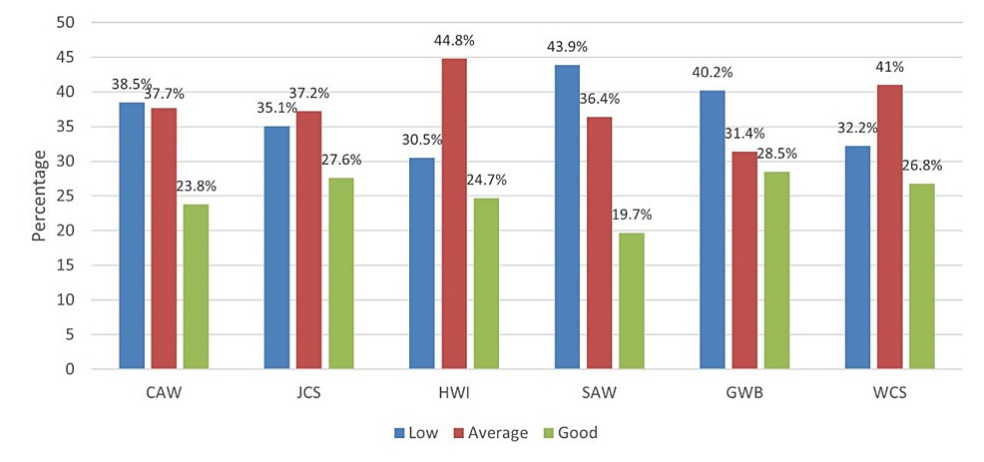
WRQoL component levels WRQoL: Work-Related Quality of Life, CAW: control at work, JCS: job and career satisfaction, HWI: home-work interface, SAW: stress at work, GWB: general well-being, WCS: working conditions

**Figure 2:**
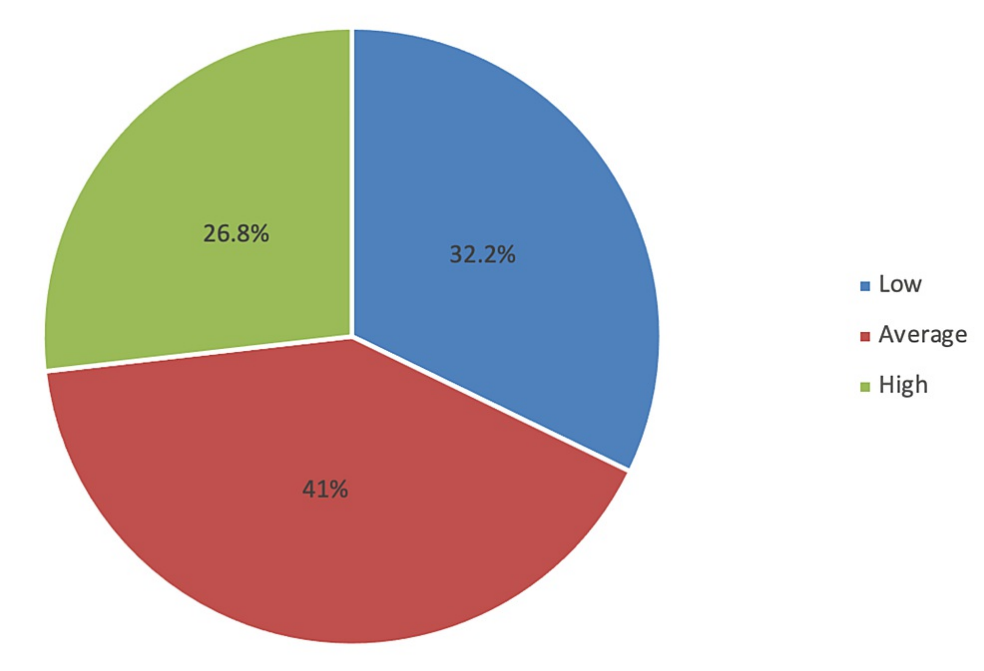
Overall WRQoL levels WRQoL: Work-Related Quality of Life

In Table 2, among the WRQoL components, HWI (mean score: 9.88 out of 15 points) and GWB (mean score: 20.6 out of 30 points) had average ratings. Other WRQoL components had good ratings, including CAW (mean score: 11 out of 15 points), JCS (mean score: 22.1 out of 30 points), SAW (mean score: 6.85 out of 10 points), and WCS (mean score: 10.8 out of 15 points) (Figure 1). The overall WRQoL rating was presumed as good, with a mean score of 81.3 out of 115 points (Figure 2).

**Table 2:** Level of WRQoL and its component (n=239) WRQoL: Work-Related Quality of Life, CAW: control at work, JCS: job and career satisfaction, HWI: home-work interface, SAW: stress at work, GWB: general well-being, WCS: working conditions

WRQoL components	Low (number (%))	Average (number (%))	Good (number (%))	Mean	Interpretation
CAW	92 (38.5%)	90 (37.7%)	57 (23.8%)	11 out of 15	Good
JCS	84 (35.1%)	89 (37.2%)	66 (27.6%)	22.1 out of 30	Good
HWI	73 (30.5%)	107 (44.8%)	59 (24.7%)	9.88 out of 15	Average
SAW	105 (43.9%)	87 (36.4%)	47 (19.7%)	6.85 out of 10	Good
GWB	96 (40.2%)	75 (31.4%)	68 (28.5%)	20.6 out of 30	Average
WCS	73 (30.5%)	112 (46.9%)	54 (22.6%)	10.8 out of 15	Good
Overall WRQoL	77 (32.2%)	98 (41.0%)	64 (26.8%)	81.3 out of 115	Good

When measuring the differences in the score of WRQoL and the sociodemographic characteristics of the residents, it was found that a higher WRQoL score was more associated with being female residents (Z=2.187, p=0.029) and having practicing residency inside central region (Z=2.674, p=0.008). No significant differences were observed between WRQoL scores in terms of residency levels, marital status, and a previous psychiatrist or psychologist visitation (p>0.05) (Table 3).

**Table 3:** Association between WRQoL and the sociodemographic characteristics of the residents (n=239) ^§^P-value calculated using Mann-Whitney Z-test **Significant at p<0.05 level WRQoL: Work-Related Quality of Life, SD: standard deviation, SCFHS: Saudi Commission for Health Specialties

Factor	WRQoL score (115) (mean ± SD)	Z-test	P-value^§^
Gender			
Male	79.7 ± 12.8	2.187	0.029**
Female	84.1 ± 15.0		
Region of training center SCFHS branch			
Inside central region	84.1 ± 13.7	2.674	0.008**
Outside central region	78.9 ± 13.3		
Residency level			
Junior residents (R1-R3)	81.2 ± 12.9	0.498	0.619
Senior residents (R4-R5)	81.6 ± 16.0		
Marital status			
Never been married	81.6 ± 13.4	0.224	0.823
Been married	80.8 ± 14.2		
Have you ever been to a psychiatrist or a psychologist before?			
Yes	83.8 ± 13.6	1.527	0.127
No	80.5 ± 13.7		

## Discussion

Our study was a survey-based investigation involving 239 general surgery residents. The main objectives of the study were twofold: to identify the quality of life among general surgery residents in Saudi Arabia and to identify the causative agents that lead to anxiety, depression, and burnout.

This study evaluates the WRQoL of general surgery residents. The overall WRQoL of residents included in this study was deemed adequate. Based on the given criteria, approximately 26.8% were considered to have good QoL, 41% were average, and 32.2% had low QoL (mean score: 81.3 out of 115 points). These findings are comparable with that of Almhizai et al. (2022) [6]. Based on their reports, more than half (51.9%) of pediatric residents demonstrated a good level of QoL, 47.7% were moderate, and only 0.4% were considered low QoL levels. A good QoL had also been detected among pediatric residents in Iran (Zare et al. (2012) [8]), with the level of QoL determined as very well, well, and moderate in 18%, 32%, and 31%, respectively. Only 19% of the residents had either low or very low QoL. Contradicting these reports, a study conducted in Jeddah (Almailabi et al. (2019) [1]) documented that around half of the residents (50.7%) working in King Abdulaziz Medical City demonstrated low WRQoL, 37% were average, and only 12.3% had high QoL.

QoL is an important indicator of residents’ motivation. However, due to workload and continuing education, residents are prone to various stressors affecting their quality of life. Hence, appropriate measures should be implemented to maintain residents’ physical and mental health leading to improved patient care.

Among the six components of WRQoL, CAW, JCS, SAW, and WCS were all classified as good levels, while HWI and GWB were classified as average levels. None of the residents were shown to have low WRQoL. In a study done in Jeddah (Almailabi et al. (2019) [1]), however, most WRQoL components were categorized into low levels, including HWI (76.7%), CAW (56.2%), GWB (47.9%), JCS (34.2%), WCS (31.5%), and SAW (31.5%). In a research done in the USA (Zubair et al. (2016) [9]), comparing the components of WRQoL between male and female residents, it was found that males were seen to have better HWI, GWB, CAW, and WCS than females, while junior residents showed lower JCS and CAW than senior residents. Furthermore, psychological disorders affect even more residents’ quality of life. For example, Alam et al. (2022) [10] reported that during the COVID-19 pandemic, 41% of surgical residents experienced more anxiety than before the pandemic, while in a paper conducted by Mordant et al. (2013) [11], 60% of the trainees had a low level of fatigue, with a little below half of them (44%) reporting having sleep disorder based on the Epworth Sleepiness Score, which is mainly due to in-hospital work time and insufficient educational programs.

Data in our study suggest that better QoL was significantly predicted in female residents who were practicing in the central region training centers. In Iran (Zare et al. (2012) [8]), a study found that urology and internal medicine residents had the lowest QoL, while pediatric residents showed the highest. On the contrary, a study conducted in Riyadh (Almhizai et al. (2022) [6]) reported no significant association between WRQoL and the sociodemographic variables of the residents, which did not coincide with our reports. On the other hand, both studies in Kuwait (Burhamah et al. (2021) [12]) and the USA (Smeds et al. (2020) [13]) noted a significant association between burnout and depression among residents, adding that burnout during residency training is detrimental to the wellness of the residents and that addressing this stressor is tantamount to improving the quality of care. According to the study by Mordant et al. (2013) [11] among residents diagnosed with depression and burnout, 20% of them recorded major medication errors during the last three months. In Dammam (Aljehani et al. (2020) [14]), a study found a link between anxiety in relation to gender, covering intensive care unit (ICU) duty, COVID-19 infection, family infected with COVID-19, and level of training. Different stressors could lead to poor quality of life, affecting their job functions. Residents are not immune to stressors. Therefore, formulating guidelines to address the impact of psychological burden in line with the residency program is crucial to the wellness of residents during their training.

One significant limitation of this study is the relatively small sample size, and other regions should be evaluated further. Consequently, the findings and conclusions derived from this study may not be representative of the larger population or accurately reflect the diversity of perspectives and experiences.

The small sample size and potential selection of the speciality bias limited the generalizability of the study’s findings and restricted the extent to which conclusions can be drawn. It is important to acknowledge these limitations, recognizing that larger, more participants would provide a more comprehensive understanding of the phenomena under investigation. Future research should aim to address these limitations by involving larger and more representative samples to ensure the validity and reliability of the study’s findings.

## Conclusions

The work-related quality of life among general surgical residents was reasonable. Better WRQoL was more prevalent among female general surgery residents of the central region training centers. However, the WRQoL between junior and senior residents was comparable in nature. This study provides evidence that the quality of life among general surgery residents in Saudi Arabia was generally satisfying. However, a multicenter study approach is necessary to shed more light on the effect of residency training on their quality of work life.

## References

[1] Almailabi MM, Alajmi RS, Balkhy AL, Khalifa MJ, Mikwar ZA, Khan MA (2019). Quality of life among surgical residents at King Abdulaziz Medical City in Jeddah, Saudi Arabia. Open Access Maced J Med Sci.

[2] (2012). World Health Organization: The World Health Organization Quality of Life (WHOQOL). https://www.who.int/publications/i/item/WHO-HIS-HSI-Rev.2012.03.

[3] van Vendeloo SN, Godderis L, Brand PL, Verheyen KC, Rowell SA, Hoekstra H (2018). Resident burnout: evaluating the role of the learning environment. BMC Med Educ.

[4] Ogawa R, Seo E, Maeno T, Ito M, Sanuki M, Maeno T (2018). The relationship between long working hours and depression among first-year residents in Japan. BMC Med Educ.

[5] Mache S, Bernburg M, Vitzthum K, Groneberg DA, Klapp BF, Danzer G (2015). Managing work-family conflict in the medical profession: working conditions and individual resources as related factors. BMJ Open.

[6] Almhizai RA, Alnazha MN, Alharbi RF, AlRumaih NA, Alkharashi AA, Albadi AK (2022). Quality of life among pediatric residents in Riyadh. Int J Community Med Public Health.

[7] Easton SA, Van Laar DL (2023). User manual for the Work-Related Quality of Life (WRQoL) scale: a measure of quality of working life. http://www.qowl.co.uk/researchers/WRQoL%20User%20manual%202nd%20Ed%20ebook%20Feb%202018%2055.pdf.

[8] Zare MH, Ahmadi B, Sari AA, Arab M, Kor EM (2012). Quality of working life on residents working in hospitals. Iran J Public Health.

[9] Zubair MH, Hussain LR, Williams KN, Grannan KJ (2017). Work-related quality of life of US general surgery residents: is it really so bad?. J Surg Educ.

[10] Alam P, Salimi A, ElHawary H, Sioufi K, Papanastasiou C, Thibaudeau S (2022). The effects of COVID-19 on Canadian surgical residents' education and wellness. Can Med Educ J.

[11] Mordant P, Deneuve S, Rivera C (2014). Quality of life of surgical oncology residents and fellows across Europe. J Surg Educ.

[12] Burhamah W, AlKhayyat A, Oroszlányová M, Jafar H, AlKhayat A, Alabbad J (2021). The predictors of depression and burnout among surgical residents: a cross-sectional study from Kuwait. Ann Med Surg (Lond).

[13] Smeds MR, Janko MR, Allen S (2020). Burnout and its relationship with perceived stress, self-efficacy, depression, social support, and programmatic factors in general surgery residents. Am J Surg.

[14] Aljehani YM, Othman SA, Telmesani NK (2020). Stress and psychological resilience among general surgery residents during COVID-19 pandemic. Saudi Med J.

